# Deep learning-based method for grading histopathological liver fibrosis in rodent models of metabolic dysfunction-associated steatohepatitis

**DOI:** 10.3389/fmed.2025.1629036

**Published:** 2025-07-04

**Authors:** Soo Min Ko, Jae-ik Shin, Yiyu Hong, Hyunji Kim, Insuk Sohn, Ji-Young Lee, Hyo-Jeong Han, Da Som Jeong, Yerin Lee, Woo-Chan Son

**Affiliations:** ^1^Department of Medical Science, AMIST, Asan Medical Center, University of Ulsan College of Medicine, Seoul, Republic of Korea; ^2^Department of R&D Center, Arontier Co., Ltd., Seoul, Republic of Korea; ^3^Department of Radiation Oncology, Yonsei University College of Medicine, Seoul, Republic of Korea; ^4^Department of Pathology, Asan Medical Center, University of Ulsan College of Medicine, Seoul, Republic of Korea

**Keywords:** artificial intelligence, deep learning, metabolic dysfunction-associated steatohepatitis, liver fibrosis, histopathology

## Abstract

**Introduction:**

Metabolic dysfunction-associated steatohepatitis (MASH) is a significant liver disease that can lead to cirrhosis and liver cancer. Accurate assessment of liver fibrosis is crucial for diagnosis, prognosis, and informed treatment decision-making. Staging of liver fibrosis in MASH is based on Kleiner’s score, which categorizes fibrosis based on its location within the liver as observed microscopically. This scoring system is part of a standard clinical research network and relies heavily on the expertise of pathologists.

**Methods:**

This study utilized Sirius Red-stained whole slide images of liver tissue obtained from various MASH animal models to develop deep learning (DL) models for scoring liver fibrosis, with a focus on the criteria outlined in Kleiner’s score. We created a trainable and testable dataset of whole-slide images of the liver, consisting of 999,711 patch images derived from 914 whole-slide images. The performance of the multi-class classification model was evaluated using the kappa statistic, area under the precision-recall curve (AUPRC), area under the receiver operating characteristic curve (AUROC), and Matthews correlation coefficient (MCC).

**Results:**

To address challenges in clinical subclassification, a 5-class classification model was initially applied; the model achieved moderate agreement. A more refined 7-class model was subsequently developed, which outperformed the 5-class classification model. The enhanced subclassification significantly improved classification performance, as evidenced by the superior AUROC and AUPRC values of the 7-class model.

**Discussion:**

This study highlights that DL models for scoring liver fibrosis can support expert pathologists in staging liver fibrosis in preclinical animal studies.

## 1 Introduction

Metabolic dysfunction-associated steatohepatitis (MASH), previously referred to as nonalcoholic steatohepatitis (NASH), is a subtype of metabolic dysfunction-associated steatotic liver disease (MASLD), also known as nonalcoholic fatty liver disease. These conditions can develop despite the absence of significant alcohol consumption. MASH is recognized as the hepatic manifestation of metabolic syndrome due to its association with insulin resistance, obesity, type II diabetes, and hyperlipidemia (1). Although liver steatosis is often not severe, approximately 25% of patients with MASH may progress to chronic cirrhosis, which can ultimately lead to hepatocellular carcinoma or liver cancer. In the United States, the incidence of MASLD-related cirrhosis between 2006 and 2010 was approximately twice as high as that of chronic hepatitis C (2).

Although MASLD refers to nonalcoholic fatty liver disease, MASH specifically denotes a distinct histological pattern of liver disease. MASH is the most common form of histologically advanced MASLD, typically involving a certain degree of fibrosis (1). It is characterized by hepatocellular ballooning, lobular inflammation, and steatosis, with or without fibrosis. The progression of fibrosis to cirrhosis in MASH is slow and unpredictable; however, advanced fibrosis is associated with an increased risk of liver-related morbidity and mortality, as well as serving as a major driver of cardiovascular comorbidity (3). Drugs targeting liver fibrosis in MASH may improve mortality independently of reducing the incidence of liver-related diseases (4).

The Food and Drug Authority currently recommends that sponsors focus drug development on non-cirrhotic MASH with fibrosis, an area of significant potential impact on human health and one of the greatest unmet medical needs (5). MASH pathology does not completely overlap between humans and mice due to differences in genetic or protein profiles. However, histopathology-confirmed consistent fibrosis in obese MASH mouse models has relatively high clinical translatability to humans. Accordingly, MASH mouse models are increasingly used for the preclinical efficacy evaluation of liver histological responses to test articles, as human MASH is highly reproducible in mouse models of MASLD (3). Biochemical parameters of the liver, histopathological scoring of liver sections by experienced pathologists using the MASH Clinical Research Network (CRN) system, and quantitative analysis of liver sections are frequently used to assess efficacy in MASLD/MASH. However, although the histopathological scoring method is globally recognized and widely adopted, it relies on subjective interpretation by expert pathologists, rendering the results subjective, time-consuming, and susceptible to interobserver variation among different pathologists (6).

Lee et al. (7) and other pathologists showed that the primary histological characteristic of MASH is the presence of fibrosis in liver biopsy specimens. Based on Kleiner’s CRN scoring system, three fibrosis subclasses (i.e., scores 1A, 1B, and 1C from score 1) can be considered as major criteria, combined with inflammation and steatosis, to diagnose “not-MASH,” “borderline,” or “MASH.” In MASLD, fibrosis typically begins with deposition around the central veins, presenting as a centrilobular or perisinusoidal pattern, which corresponds to stage 1A or 1B fibrosis. As the improvement, stabilization, and progression of fibrosis are major endpoints in the transition from MASLD to its progressive form, MASH, accurate staging of fibrosis based on its architecture is essential (8). Several digital pathology techniques utilizing computer software have been used to quantify fibrosis (9–13). Masson’s trichrome and Sirius Red are the most commonly used histochemical stains that highlight collagen (14) and are used to assess the extent of staining. Both Masson’s trichrome and Sirius Red have been used for computer-assisted morphometric analysis of liver fibrosis. However, Sirius Red demonstrates superior performance due to its higher sensitivity in detecting early-stage perivascular or pericellular fibrosis (15, 16), which is particularly useful for staging fibrosis based on Kleiner’s CRN scoring system. Further, Masson’s trichrome requires careful optimization to prevent over- or understaining, which can compromise the evaluation of fibrosis. In contrast, Sirius Red provides consistent and interpretable results without the need for extensive protocol optimization (17). However, accurate analysis of individual structural components remains unattainable (9). ImageJ (National Institutes of Health, MD, USA), one of the simpler digital pathology methods (13, 18), does not conform to the criteria of Kleiner’s scoring system as it solely quantifies the amount of fibrosis. Furthermore, the representativeness of the entire slide is questionable, given that it only analyzes a randomly assigned region of interest selected by the analyst rather than the whole-slide image (WSI). Analyzing a sufficiently large number of regions of interest to replace the WSI on a single slide would be time-consuming. In contrast, the deep learning (DL)-based model we have developed is designed to analyze the WSI and classify fibrosis based on a standardized scoring system.

Farzi et al. (19) has introduced Liver-Quant, an open-source Python-based software, for quantifying fibrosis using Masson’s trichrome-, Sirius Red-, and Van Gieson-stained WSI in MASLD. Liver-Quant measures the collagen proportionate area (CPA) based on the morphological features and staining color to estimate the extent of fibrosis. The CPA values demonstrate a moderate correlation with pathologist assessment. However, CPA has limited capability to provide detailed insights into the morphological and pathophysiological aspects of liver fibrosis as it does not reflect the liver architecture or the spatial distribution of fibrosis. Furthermore, the substantial overlap in CPA values across different semi-quantitative fibrosis stages may reduce its accuracy in grading fibrosis severity.

Recent studies have designed and validated digital pathology and DL-based methods to quantify fibrosis and other histological features of MASH, including ballooning degeneration, lobular inflammation, and steatosis (13, 18, 20–22). In these studies, ballooning degeneration, lobular inflammation, steatosis, and fibrosis were scored based on the extent of the affected area in liver sections, which could be effectively quantified using DL methods. For fibrosis, Heinemann et al. (21) suggested that distinguishing between fibrosis scores 0 and 1 using CPA-based analysis can be challenging as early-stage fibrosis does not significantly alter the collagen-stained area. In contrast, artificial intelligence-based analysis can detect subtle fibrotic alterations, successfully differentiating score 1 from score 0. However, Kleiner’s fibrosis scoring system is based on the amount of fibrosis and its microanatomical location. Gawrieh et al. (8) developed DL-based methods that incorporated CPA-based quantification alongside annotations of fibrosis architectural patterns, including perisinusoidal, periportal, bridging, and nodular fibrosis. This model showed high accuracy in detecting its liver architecture and quantifying fibrosis. However, the model had more challenges detecting perisinusoidal and periportal patterns (corresponding to stage 1 fibrosis) than bridging or nodular patterns (corresponding to stage 3 or 4 fibrosis, respectively). This reflects real-world pathological practice where advanced fibrosis is more readily recognized and has higher inter- or intra-observer consistency.

Although these studies have successfully quantified fibrosis, none of them have used a standardized scoring classification method that subdivides a fibrosis score of 1 into scores 1A, 1B, and 1C, which are based on the location of the affected fibrosis. Hence, the current study focused on DL-based fibrosis quantification, specifically subclassifying a score of 1 into scores 1A, 1B, and 1C, which could ultimately be applied in both preclinical and clinical settings.

## 2 Materials and methods

### 2.1 Animal models

Liver tissue sections from previous animal studies conducted between 2018 and 2023 were reanalyzed. C57BL/6 J mice and Wistar rats of various ages were obtained from OrientBio (Seongnam, Korea) and Charles River (Sulzfeld, Germany). The animals were maintained in accordance with the guidelines set forth by the Institutional Animal Care and Use Committee of Asan Medical Center (IACUC number 2019–14-123, 2020–02-234 and 2021–02-029) and other facilities. The disease models used included established methods, such as carbon tetrachloride, thioacetamide, choline-deficient L-amino acid-defined high-fat diet (HFD), a methionine-choline deficient-HFD, and streptozotocin-induced hepatitis (STAM). These methods resulted in varying degrees of morphological changes that correlated with MASH. The methionine-choline deficient-HFD mouse model was induced by feeding a diet containing 40% sucrose and 10% fat without methionine and choline (Research Diets, A02082002BR) for 8 weeks. The STAM mouse model was induced by a single subcutaneous injection of 200 μg streptozocin (S0130, Sigma-Aldrich, MO, USA) 2 days after birth, followed by feeding with a 60 kcal% fat diet (Research Diets, D12492) starting at age 4 weeks. Liver slides were obtained from different facilities that had established carbon tetrachloride, thioacetamide, and CDA-HFD MASH mouse models for their respective efficacy studies.

### 2.2 Histopathological examination

The animals were housed at Asan Medical Center and euthanized under isoflurane-induced anesthesia. Liver tissues obtained during necropsy were preserved in 10% neutral-buffered formalin for over 24 h. The tissues were routinely processed, embedded in paraffin, sectioned, and stained using the Picrosirius Red stain kit (24901–500, Polysciences, Inc., PA, USA) before being examined microscopically. In addition, Sirius Red-stained slides of carbon tetrachloride, thioacetamide, and choline-deficient L-amino acid-HFD mice models were obtained from other facilities. WSIs of liver sections intended for DL-based analysis were scanned using the Motic EasyScan Pro 6 (Motic, Vancouver, Canada). Histopathological scoring was conducted by an experienced veterinary pathologist utilizing the MASH CRN system (Table 1).

**Table 1:** Fibrosis histopathological scoring system based on Kleiner et al. (23).

Score	Definition
0	None
1A	Mild, zone 3, perisinusoidal
1B	Moderate, zone 3, perisinusoidal
1C	Portal/periportal
2	Zone 3, perisinusoidal and portal/periportal
3	Bridging fibrosis
4	Cirrhosis

### 2.3 DL-based approach for fibrosis stage scoring

This study proposes two DL models for classifying stages of liver fibrosis. The first model categorizes fibrosis into 5 classes based on a scoring system (0, 1, 2, 3, or 4). The second model further subdivides score 1 into 7 subclasses (0, 1A, 1B, 1C, 2, 3, or 4). These subclasses are determined by histological differences observed in the central vein, portal triad areas, and fibrosis patterns, which are known to be clinically challenging to differentiate. Our approach offers the advantages of reproducibility and quantification in classification, adhering to the clinically established fibrosis stages for patients with MASH (23).

### 2.4 Patch-wise image preprocessing

All WSIs were saved in Aperio format (SVS) and imported into OpenSlide Python for subsequent processing (24). Due to the large size of WSIs, a set of non-overlapping patches measuring 1,024 × 1,024 pixels was extracted from a WSI scanned at 40 × magnification. To maintain consistent physical dimensions of tissue between 20 × and 40 × scanning magnifications, patches measuring 512 × 512 pixels were extracted from a WSI scanned at 20 × magnification. The patches, each measuring 0.27 × 0.27 mm^2^, were utilized in training the classification model. Patch-wise preprocessing was conducted to exclude background patches and resize the selected patches. To eliminate background patch images, the patch images were converted to grayscale and binarized to differentiate between tissue and background using manual thresholding of pixel values. Only those patch images containing at least 75% tissue were used for training and validation. A set of WSI patch images was selected, as described above. To utilize computing resources efficiently, the selected WSI patch images were downsampled to 256 × 256 pixels. Ultimately, the dataset comprised 999,711 patch images derived from 914 WSIs.

### 2.5 Class distribution and dataset splitting

The distribution of liver fibrosis scores from 917 WSIs is provided in Table 2. Each score category contained over 100 WSIs, with score 0 comprising 20% of the entire dataset. The score 1 subclass accounted for over 40% of the total, including more than 100 WSIs for each subclass. This allowed for individual evaluation of subclass classification. A 5-fold cross-validation was conducted to assess our methodology. The complete set of WSIs was divided into five subsets, each maintaining the same distribution of scores as the overall dataset. One subset was designated for validation, while the remaining four subsets were utilized for training the model. This process was repeated five times, with different training and validation sets in each iteration.

**Table 2:** WSI dataset and patch images divided by score.

Score	0	1			2	3	4	Total
		1A	1B	1C				
Number of WSIs	187	146	121	113	133	108	106	914
Ratio of WSI	20.5%	16.0%	13.2%	12.4%	14.6%	11.8%	11.6%	100%
Number of patch images	173,598	116,566	171,051	74,433	166,974	148,739	148,350	999,711
Ratio of the patch image	17.4%	11.7%	17.1%	7.5%	16.7%	14.9%	14.8%	100%
			36.2%					

WSI, whole-slide image.

### 2.6 Patch-level model training

**Figure 1:**
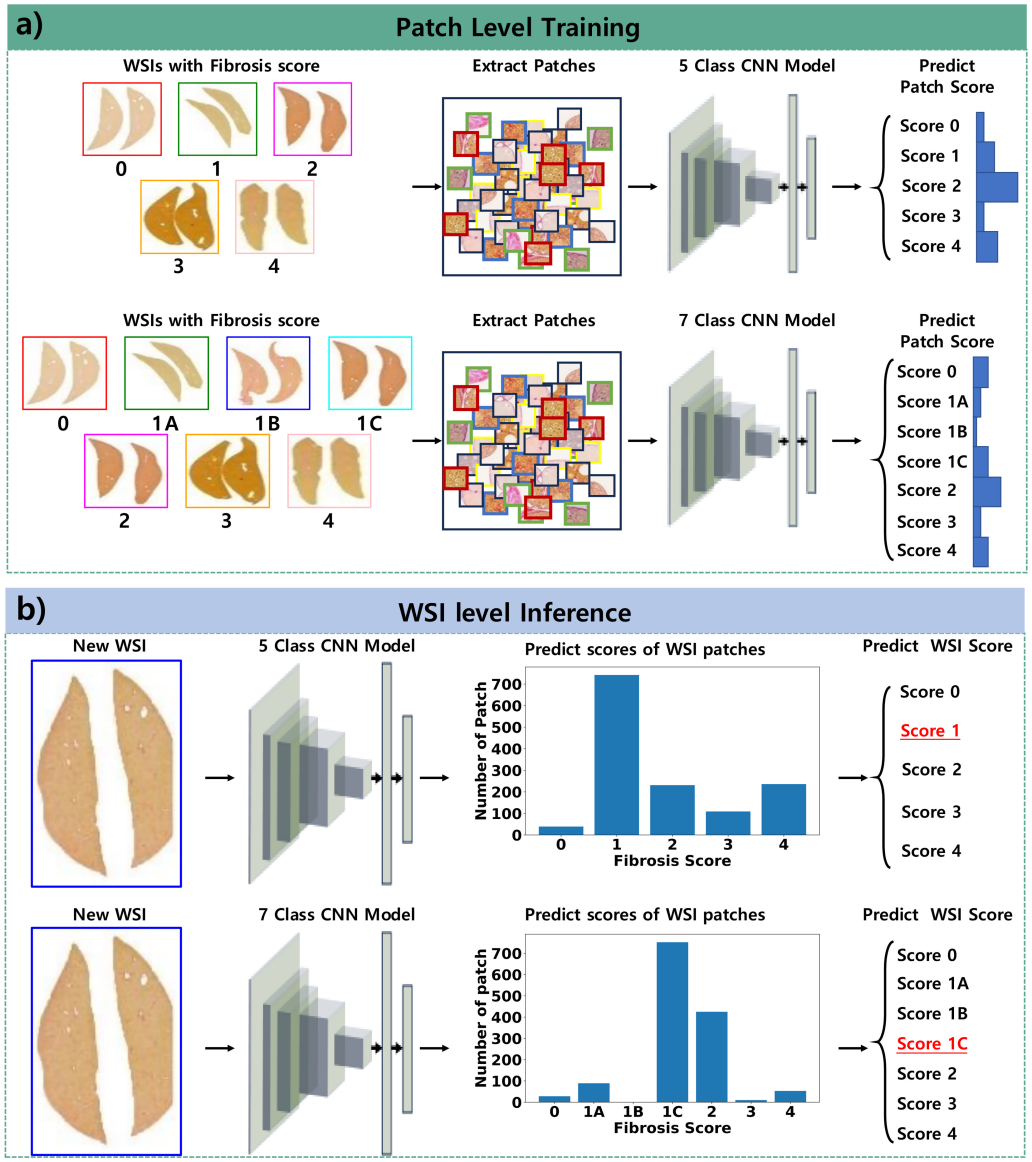
Pipeline of the proposed approach for classifying fibrosis stages. **(a)** Patch-level training involves collecting patches from WSIs and training the model to classify the fibrosis score derived from these WSIs. **(b)** WSI-level inference entails collecting patches from new WSIs, predicting the scores of these patches, and determining the overall fibrosis score as the most frequently occurring score among the patches within the WSI.

Our deep convolutional neural network for multi-class classification was based on the ResNet34 architecture (25). The final layer of the network was modified to predict a fibrosis score from a 512-dimensional feature vector extracted by the residual layers from a patch image. The patch images were assigned labels corresponding to fibrosis scores derived from WSIs (Figure 1a).

The network is trained to reduce a cross-entropy loss between the predicted score and the true score of the patch:


$$L_{n=0\sim N}(x,y)=-\sum_{j=1}^{C}[w_{y}\log\frac{e^{x_{n,j}}}{\sum_{i=1}^{C}e^{x_{n,i}}}y_{n,j}]$$

where ***x*** is the predicted class, ***y*** is the true class, ***C*** is the number of classes, ***w*** is the weight of the class, and **N** is the number of patches in a minibatch. Due to the differing score distributions between patches and WSIs, class weights were applied to calculate the loss during training. These weights were based on the score distribution from the patches in the training set (Table 2). The weight for each class is determined using the inverse of the number of samples as follows:


$$w_{i}=\frac{1}{2}\frac{\sum_{j}^{C}N_{j}}{N_{i}}$$

The RAdam optimizer (26) was used to update the network using mini-batches of 128 patches, with a learning rate set at 0.001, beta set at 0.9–0.999, and epsilon set at 1e-8. Model training was iterated until the Matthews correlation coefficient (MCC) and Kappa from the validation results exceeded 0.8, completing the process within 50 epochs. In addition, image preprocessing and data augmentation techniques were applied to these tissue patches during training. Image preprocessing of the patches was performed using the heuristic pixel thresholding in Hue, Saturation, and Value color space to highlight collagen fibers stained by Picro Sirius Red (19). Data augmentations on the preprocessed images were performed. These augmentations included a combination of affine transformations (e.g., random rotation, horizontal and vertical flipping) and color space manipulations (e.g., random changes in brightness and contrast; random gamma correction; and random adjustments to hue, saturation, and value). Finally, the image was normalized to a value between 0 and 1 for use as input for training the model.

### 2.7 Hyperparameter tuning

To assess the influence of hyperparameter settings on model performance, a limited grid search was conducted, focusing primarily on the learning rate of the RAdam optimizer. Two learning rates, 1e-3 and 1e-4, were evaluated while keeping other optimizer parameters fixed. Each configuration was trained for 100 epochs using the training and validation datasets, and performance was measured using the MCC and Cohen’s Kappa score.

A learning rate of 0.001 combined with 50 training epochs was ultimately selected, as this configuration consistently yielded MCC and Kappa values exceeding 0.8 while maintaining stable convergence and minimizing overfitting. Additionally, the impact of patch size was explored as a secondary factor. Input patches of 1,024 × 1,024 pixels and 2,048 x 2,048 pixels were both resized to 256 × 256 pixels before being fed into the model. Among these, the model trained on 1,024 × 1,024 patches demonstrated superior performance with respect to MCC and Kappa, suggesting that this resolution preserved sufficient contextual and structural information to support accurate classification under consistent training conditions.

### 2.8 WSI-level model inference

The models described above were validated using 5-fold cross-validation with WSIs from the validation dataset. After aggregating all patch predictions for a WSI, the most frequently predicted class was designated as the fibrosis stage for that WSI (Figure 1b).

The identical image preprocessing applied during model training was applied to the validation dataset, except for data augmentation.

The performance of the proposed models was evaluated using metrics commonly used in multi-class classification tasks. Cohen’s Kappa coefficient (Kappa) indicated the level of agreement between the predicted and actual fibrosis scores for WSIs, with a value >0.75 signifying excellent agreement (25). Additionally, linear- and quadratic-weighted Kappa values were considered, as the agreement in higher fibrosis score classifications was deemed more significant. In comparison to accuracy, MCC offers a more informative evaluation in scenarios with class imbalance (27, 28).

The receiver operating characteristic curve illustrated the relationship between the true positive rate and the false positive rate, while the precision-recall curve depicted the relationship between precision and recall. Both curves were generated using the ratio of predicted classes from patches of WSIs. For multi-class classification, the area under the receiver operating characteristic curve (AUROC) and area under the precision-recall curve (AUPRC) were calculated using the one-vs-rest technique, comparing WSIs with the target fibrosis score against those with other scores. This strategy transformed a multi-class classification into a binary classification for each class. It was determined that a designated class label would be positive while all other class labels would be negative. Finally, the average AUROC and AUPRC were computed to provide a comprehensive evaluation across all classes and folds. The AUPRC is particularly sensitive to class imbalances between the dataset of the target class and the other classes.

## 3 Results

The proposed models were trained to predict fibrosis scores ranging from 0 to 4 for WSIs. The results are detailed in Tables 3, 4. As a result of 5-fold cross-validation, the models were trained and validated for a maximum of 50 epochs each. The best validation performance was achieved at the 37th epoch for the 5-class classification model and at the 34th epoch for the 7-class model.

**Table 3:** Result of the 5-class classification model.

Fold	Kappa	L-Kappa	Q-Kappa	MCC	AUROC	AUPRC
0	0.815	0.873	0.912	0.815	0.982	0.949
1	0.869	0.909	0.937	0.871	0.979	0.927
2	0.793	0.785	0.770	0.794	0.968	0.914
3	0.732	0.784	0.812	0.735	0.941	0.826
4	0.808	0.851	0.880	0.810	0.959	0.913
Average	0.803	0.840	0.862	0.805	0.966	0.906

AUROC, area under the receiver operating characteristic curve; AUPRC, area under the precision-recall curve; L-Kappa, linear Kappa; MCC, Matthews correlation coefficient; Q-Kappa, quadratic Kappa.

**Table 4:** Result of the 7-class classification model.

Fold	Kappa	L-Kappa	Q-Kappa	MCC	AUROC	AUPRC
0	0.801	0.814	0.820	0.803	0.981	0.916
1	0.814	0.850	0.877	0.817	0.985	0.929
2	0.808	0.822	0.819	0.811	0.973	0.904
3	0.807	0.861	0.895	0.809	0.984	0.930
4	0.806	0.869	0.908	0.807	0.967	0.887
Average	0.807	0.843	0.864	0.809	0.978	0.913

AUROC, area under the receiver operating characteristic curve; AUPRC, area under the precision-recall curve; L-Kappa, linear Kappa; MCC, Matthews correlation coefficient; Q-Kappa, quadratic Kappa.

**Figure 2:**
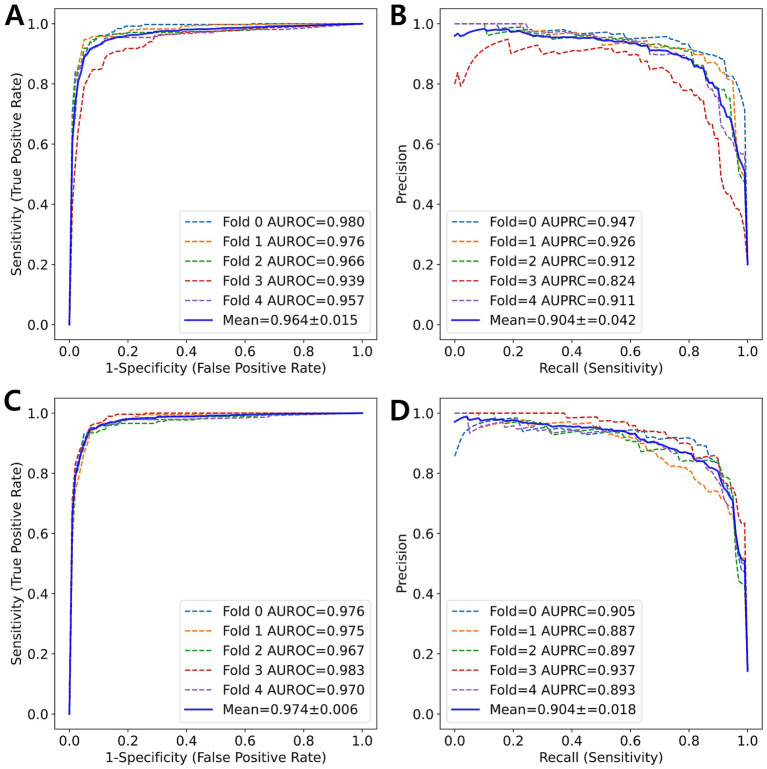
ROC curve and PR curve (blue solid line) represent the averaged curves from each fold (colored dotted lines) obtained through 5-fold cross-validation. **(A)** ROC curve and **(B)** PR curve for 5-class classification. **(C)** ROC curve and **(D)** PR curve for 7-class classification.

Using 5-fold cross-validation, the 5-class model achieved an average Kappa of 0.803, indicating moderate agreement between the predicted and true fibrosis scores. The weighted Kappa values (linear: 0.840, quadratic: 0.862) suggested a higher level of agreement. An MCC of 0.805 indicated good performance for classification tasks despite a score of 1, which constituted over 40% of the entire dataset. By averaging the results from the one-vs-rest technique for each fold, the average AUROC was 0.966, demonstrating excellent performance. Additionally, the AUPRC was 0.906, which accounted for the class imbalance between the target scores and others. The 7-class model achieved unweighted, linear, and quadratic values of 0.807, 0.843, and 0.864, respectively; an MCC of 0.809; AUROC of 0.978; and AUPRC of 0.913, showing slightly superior performance compared to the 5-class model. Figure 2 illustrates the ROC curve and PR curve from the 5-fold cross-validation process.

**Figure 3:**
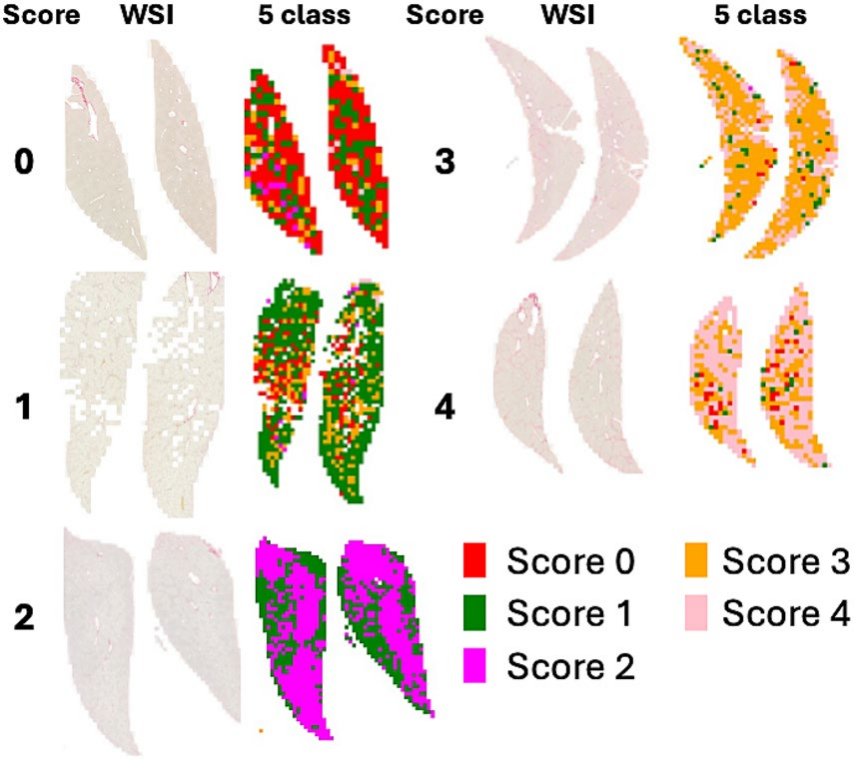
WSI and heatmaps for fibrosis scores (0, 1, 2, 3, and 4). The scores for the patch images derived from WSI are predicted using a 5-class classification model, and the scores for the patches are represented on a color map.

Figure 3 shows a heatmap visualization of the predicted scores (0, 2, 3, 4) for various patches extracted from a WSI. Each color in the heatmap corresponds to a specific score predicted by the model for a particular image patch. The most frequent score is visually emphasized by a distinct color in the heatmap.

**Figure 4:**
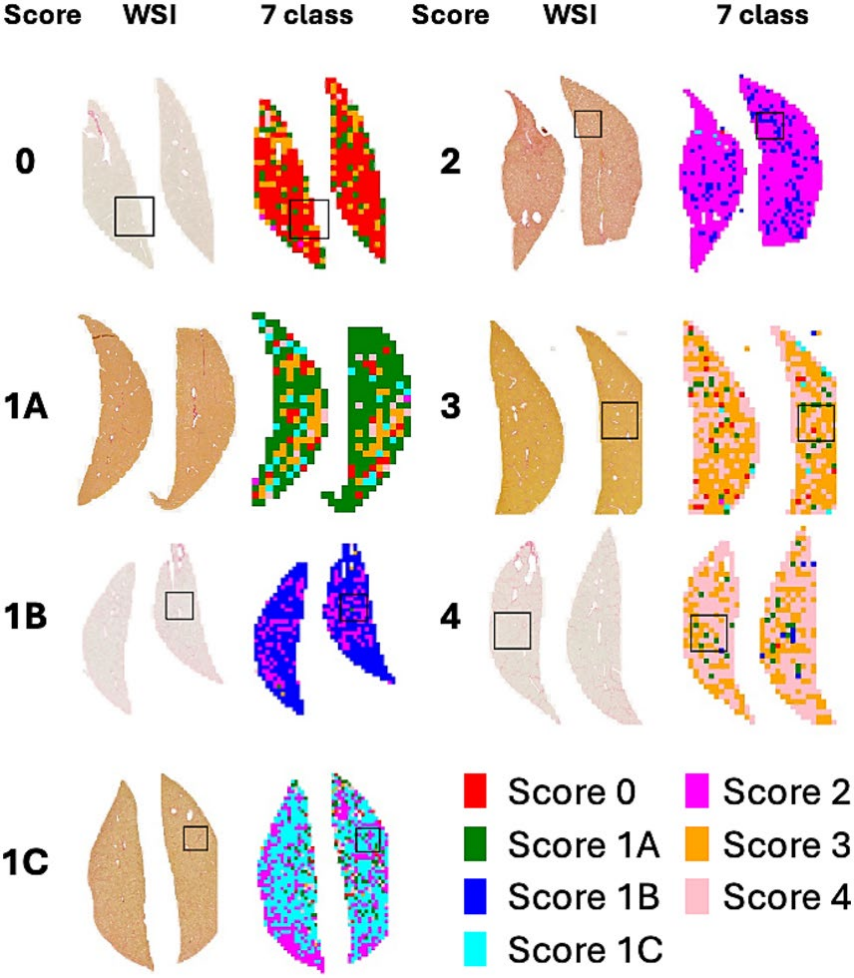
WSI and heatmaps for fibrosis scores (0, 1A, 1B, 1C, 2, 3, and 4). The scores for the patch images derived from WSI are predicted using a 7-class classification model, and these scores are mapped onto a color gradient. To illustrate the process of patch-level inference, a specific area in the image and heatmap is selected for closer examination in Figure 5.

**Figure 5:**
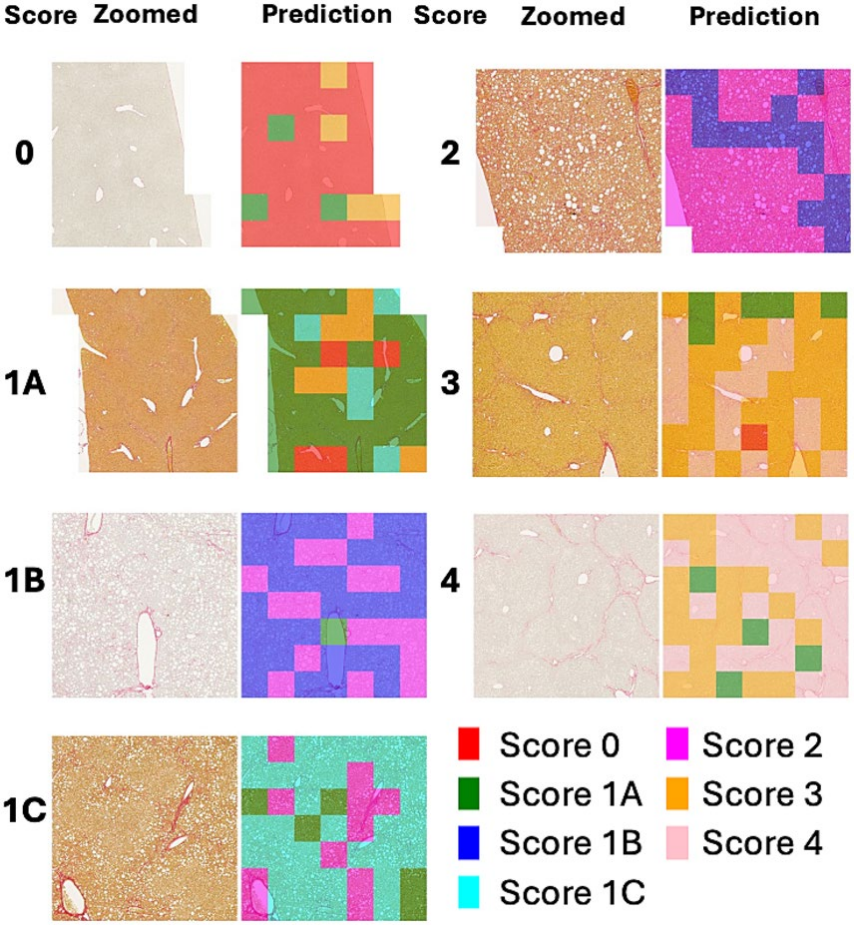
Example of patch-level inference results from the 7-class classification model. The original patches (left: zoomed) are selected from a box of WSIs in Figure 4. Background patches are excluded due to the absence of tissue. The patch-level inference results are mapped onto the patches using a color map (right: predictions).

Figure 4 shows a heatmap comparing the scores of 1 with subclasses (1A, 1B, and 1C). Figure 5 illustrates the results of patch-level inference using a 7-class classification model. The patches were extracted from a box of WSIs, as shown in Figure 4. Each image displays the original patches (left) and the color-overlayed patches (right). Background patches were excluded from the inference process.

## 4 Discussion

Liver fibrosis is a crucial parameter for diagnosing, evaluating prognosis, and assessing drug responsiveness in chronic liver diseases. Grading liver fibrosis in biopsy specimens is considered the gold standard in both clinical practice and animal models of MASH (29–32). In a retrospective study of patients with MASLD, among the histological features of the disease, only the fibrosis stage was directly associated with overall mortality and prognosis (33). However, grading liver fibrosis has the limitation of relying on the subjective interpretation of experienced pathologists.

Heinemann et al. (6, 21) introduced a DL-based model for scoring fibrosis stages that utilized a convolutional neural network architecture to extract features from WSIs. Our approach involved modifying a convolutional neural network architecture, specifically ResNet34, which is commonly used in classification tasks. Heinemann et al. (21) applied an additional model to categorize the WSI-level score based on the feature map generated by the convolutional neural network. In the current study, the WSI-level fibrosis score was determined by identifying the most frequent scores from patches classified by our model. This method was predicated on carefully balancing the number of samples across different fibrosis stage scores to facilitate subclassification. To enhance the generalizability of the dataset, liver sections with varying staining intensities and patterns were collected from multiple testing facilities and MASH models. Furthermore, weights assigned to each class were incorporated into the calculation of the cross-entropy loss during the training process to address the imbalance in the number of patches extracted from the WSI.

The multi-class classification model was evaluated using the kappa statistic (6, 21), along with metrics derived from the confusion matrix. Hameed et al. (34) and Yu et al. (20) used the AUPRC and AUROC for each class. In the current study, the MCC and AUPRC were used to evaluate an imbalanced dataset in conjunction with the kappa statistic and AUROC. Our 5-class classification model demonstrated strong agreement.

**Figure 6:**
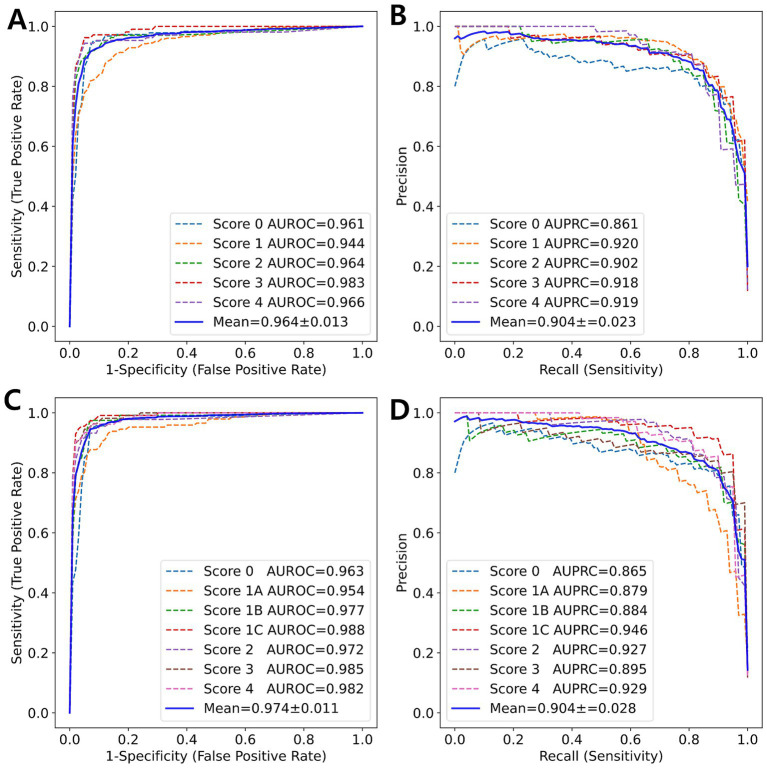
ROC curve and PR curve (blue solid line) represent the average of the curves for each class (colored dotted lines) obtained from 5-fold cross-validation. **(A)** ROC curve and **(B)** PR curve for 5-class classification. **(C)** ROC curve and **(D)** PR curve for 7-class classification.

Unlike previous studies, the current study developed a 7-class classification model to address the significant clinical challenge of distinguishing between subclasses. Our 7-class classification model exhibited superior performance compared to the 5-class classification model. The subclass classification enhanced the overall classification performance, as evidenced by the differences in AUROC and AUPRC between the two models (Figure 6).

The heatmaps of WSIs for fibrosis scores displayed mixed-colored patches rather than a uniform color. This variation suggests that multiple fibrosis features may coexist within a single liver tissue sample, potentially leading to inter-observer variability among pathologists. An example of patch-level inference is illustrated in Figure 5. In score 1A, mild fibrosis is evident in the central vein. A score 0 patch, indicating an area with no fibrosis, could be seen around the portal. However, score 1C or score 3 patches were also observed, representing periportal fibrosis or bridging fibrosis, respectively, likely due to false-positive predictions. Furthermore, as depicted in Figure 5, score 1C shows collagen fibers extending from certain portal tracts or central veins, which may be classified as score 2 or as colored patches corresponding to scores 1A or 1B. These collagen fibers are normal structures found in healthy blood vessels that have been stained with Sirius Red and can be misinterpreted as fibrosis. However, in cases with a severe degree of MASH progression, ballooning degeneration and lipid droplets associated with steatosis can be mistaken for blood vessels, causing score 1B patches to appear. Moreover, liver fibrosis initially develops around the central vein and subsequently extends to the portal tract due to their physiological characteristics (35). In advanced stage of fibrosis, fibrotic changes around the central vein and portal areas are often intermingled, such that score 1B and score 2 patches are frequently observed together. Similarly, score 3 exhibits a score 0 patch in an area devoid of bridging fibrosis. In score 2, fibrosis is present in both the central vein and portal tract, and the prediction of colored patches aligns with the pathologist’s score. Score 4 is predicted as patches that are consistent with the pathologist’s score due to the characteristic appearance of liver cirrhosis; however, certain areas exhibit attenuated fibrosis resembling bridging fibrosis, which are predicted as score 3.

**Figure 7:**
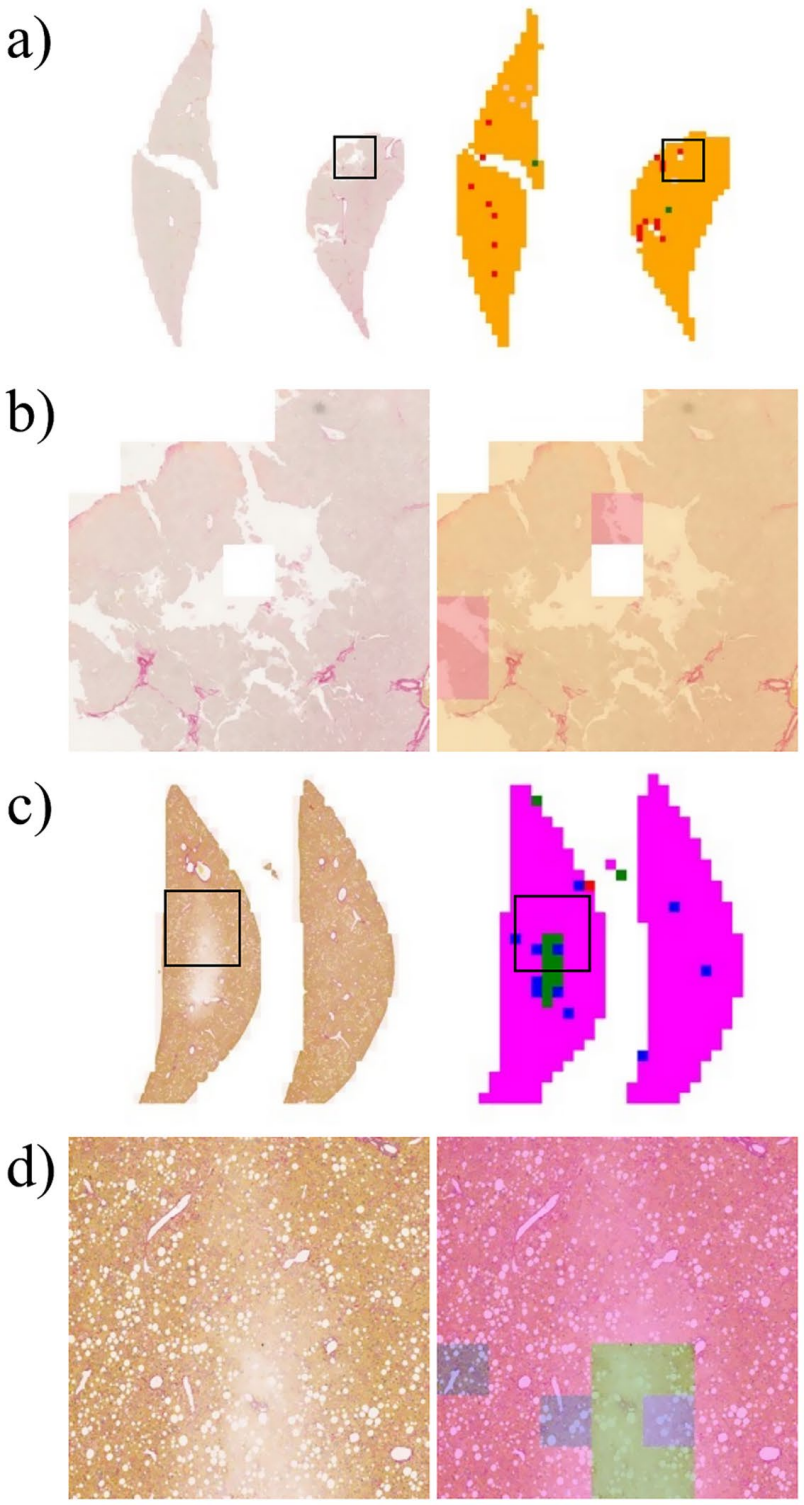
Example of error in patch-level inference due to artifacts that occur during the preparation of the liver slide. **(a)** Left: original WSI; right: WSI-level inference result. **(b)** Zoomed image from a section of the image in Panel **a**. **(c)** Left: original WSI; right: WSI-level inference result. **(d)** Zoomed image from a section of the image in Panel **c**.

Artifact is a broad term that refers to alterations in the components of tissue structure caused by extraneous factors, such as biopsy, fixation, processing, sectioning, and staining. These factors can lead to improper tissue preparation (35). Figure 7 shows overstaining artifacts leading to false-positive fibrosis predictions. Experienced pathologists often exclude these artifacts when reading and scoring slides; however, DL algorithms may misrepresent artifacts in inaccurately scored heatmaps, which can adversely affect overall score predictions.

A previous study (36, 37) attempted to detect and identify these potential artifacts in the inference process. The study focused on the detection of artifacts present on the slide, with the understanding that hematoxylin and eosin-stained slides were typically used in such cases. Future studies must consider incorporating an artifact detection process into the preprocessing pipeline. The challenge is to collect artifacts on Sirius Red-stained slides. Furthermore, an artifact dataset from a hematoxylin and eosin slide can be converted into a universal artifact dataset by applying stain normalization between different stain protocols.

Further research may be necessary to develop a pathologist-like scoring or staging system for DL, which should include additional annotations of vessels, portal tracts, bile ducts, and artifacts that can result in inaccurate predictions. Moreover, the clinical application of our study is limited because we have exclusively focused on DL methods for detecting fibrosis. The integration of DL methods for assessing ballooning degeneration, lobular inflammation, and steatosis is expected to be widely adopted in the routine pathological grading of MASLD/MASH (21). Despite these limitations, our study is significant in subdividing fibrosis score 1 into advanced stages, which may enhance the assessment efficacy of MASH treatments in preclinical studies, particularly those targeting fibrosis, and assist specialized pathologists in their evaluations.

## Data Availability

The original contributions presented in the study are included in the article/supplementary material, further inquiries can be directed to the corresponding author.
